# Trends in data quality and quality indicators 5 years after implementation of the Dutch Hip Fracture Audit

**DOI:** 10.1007/s00068-022-02012-y

**Published:** 2022-06-13

**Authors:** F. S. Würdemann, P. Krijnen, E. W. van Zwet, A. J. Arends, M. J. Heetveld, M. C. Trappenburg, J. H. Hegeman, I. B. Schipper, The Dutch Hip Fracture Audit Group, A. H. Calf, P. W. van Egmond, M. van Eijk, M. van Heijl, C. Luyten, B. G. Schutte, S. C. Voeten

**Affiliations:** 1 Department of Trauma Surgery, Leiden University Medical Center, 2333 AA Leiden, The Netherlands; 2 Dutch Institute for Clinical Auditing, Scientific Bureau, Leiden, The Netherlands; 3 Department of Trauma Surgery, Leiden University Medical Center, Leiden, The Netherlands; 4 Department of Biomedical Data Sciences, Leiden University Medical Center, Leiden, The Netherlands; 5 Department of Geriatrics, Maasstadziekenhuis Rotterdam, Rotterdam, The Netherlands; 6 Department of Trauma Surgery, Spaarne Gasthuis, Haarlem, The Netherlands; 7 Department of Internal Medicine, Ziekenhuis Amstelland, Amstelveen, The Netherlands; 8 Department of Trauma Surgery, Ziekenhuisgroep Twente, Almelo, The Netherlands

**Keywords:** Hip fractures, Quality indicators, Clinical auditing, Registries

## Abstract

**Purpose:**

The Dutch Hip Fracture Audit (DHFA), a nationwide hip fracture registry in the Netherlands, registers hip fracture patients and aims to improve quality of care since 2016. This study shows trends in the data quality during the first 5 years of data acquisition within the DHFA, as well as trends over time for designated quality indicators (QI).

**Methods:**

All patients registered in the DHFA between 1-1-2016 and 31-12-2020 were included. Data quality-registry case coverage and data completeness-and baseline characteristics are reported. Five QI are analysed: Time to surgery < 48 h, assessment for osteoporosis, orthogeriatric co-management, registration of functional outcomes at three months, 30-day mortality. The independent association between QI results and report year was tested using mixed-effects logistic models and in the case of 30-day mortality adjusted for casemix.

**Results:**

In 2020, the case capture of the DHFA comprised 85% of the Dutch hip fracture patients, 66/68 hospitals participated. The average of missing clinical values was 7.5% in 2016 and 3.2% in 2020. The 3 months follow-up completeness was 36.2% (2016) and 46.8% (2020). The QI ‘time to surgery’ was consistently high, assessment for osteoporosis remained low, orthogeriatric co-management scores increased without significance, registration of functional outcomes improved significantly and 30-day mortality rates remained unchanged.

**Conclusion:**

The DHFA has successfully been implemented in the past five years. Trends show improvement on data quality. Analysis of several QI indicate points of attention. Future perspectives include lowering the burden of registration, whilst improving (registration of) hip fracture patients outcomes.

## Introduction

Health care costs associated with hip fractures are high and still increasing [1–4]. In the Netherlands, hip fracture patients make up 24% of all trauma patients with an Injury Severity Score (ISS) ≤ 15 [5]. The Dutch Trauma Registry documented 17,237 hip fracture patients in 2015 and 18,438 hip fracture patients in 2019, indicating an increase in hip fractures of 7 percent in 4 years’ time [5]. Therefore, effective and efficient treatment leading to the best achievable outcome is of even more importance [1, 4].

Clinical auditing provides a tool to assess the effectiveness and efficiency of treatment of hip fracture patients. After the start of the first hip fracture registry in Sweden in 1988, at least ten national hip fracture registries were initiated in several countries [6]. The downside of implementing a registry is the burden of administration, which may be reflected in the data quality; especially data of functional outcome during follow-up prove difficult to collect [7].

The benefits of clinical auditing have been described in several other health care domains [8, 9]. Although auditing is likely to have a positive impact on the quality of care, the evidence base is still limited for hip fracture surgery. A comparison of a snapshot study of the Scottish Musculoskeletal Audit Group and the Scottish Hip Fracture database showed a decrease in quality of care when the hip fracture specific audit was discontinued; the time that hip fracture patients stayed on the emergency ward and the time to theatre increased, and when the audit was re-introduced improvements in these waiting times were demonstrated [10]. The National Hip Fracture Database, after implementation in England, reported a decline in time to surgery and a reduced mortality from 2003 to 2011 [11]. More recently the Irish Hip Fracture database published improved adherence to the Irish Hip Fracture standards [6, 12]. These findings provide some insight into changes in quality of care that may be attributable to the implementation of nationwide registries and the quality of these care registries. Sharing hip fracture registry analyses enables (inter)national benchmarking and analysis of trends in hip fracture care, which may demonstrate the positive impact of auditing on the quality of hip fracture care [13].

In 2016, the registry of hip fracture patients in the Netherlands in the Dutch Hip Fracture Audit (DHFA), was initiated under the umbrella of the Dutch Institute for Clinical Auditing (DICA). The DHFA aims to improve the quality of care for hip fracture patients, by providing hospitals with information to mirror and benchmark their performance. Centralized registration also facilitates uniform calculation of quality indicators on hospital-level, that are mandatory and used by the Health Care Inspectorate and the National Health Care Institute. This study aims to show trends in the quality of the data within the DHFA as well as development over time and the between-hospital variation of several quality indicators for hip fracture care.

## Methods

### Data sources

Data were derived from the DHFA. Hospitals participating in the DHFA reported their data either through an online survey or per batched datasets. All adult hip fracture patients registered between 1-1-2016 and 31-12-2020 were included. Peri-prosthetic and pathological fractures are exclusion criteria for registration in the DHFA. Dates of death were derived from the Dutch Vektis data institute, which collects data from health insurance reimbursements [14]. Since 2020 the DHFA and Vektis data are joined by a trusted third party using social security numbers. The researchers were provided with a pseudonymized dataset.

### Outcome measures

Three dimensions of data quality are described, conform the recommended minimum definition of O’Reilly et al. [15]: (1) Case capture of the DHFA, defined as the number of participating hospitals per year and number of patients recorded per hospital per year, compared to the Dutch Trauma Registry. Case capture per registration year is shown as a boxplot with median, interquartile range and minimum–maximum number of patients per hospital. (2) Data completeness, which is shown as percentage of missing values per year, in total, per section (e.g. clinical, follow-up) and per variable. (3) Data accuracy, measured by linking with other data sources of the same population. In the current study, this is done by comparing DHFA data and Vektis data for dates of death. Databases were linked as described above. Data accuracy of the date of death was determined by calculating the time difference in days between the date of death entered in the DHFA dataset and the Vektis dataset for each patient. When the calculated difference did not exceed 1 day, the DHFA date of death was considered accurate.

The DHFA uses a set of quality indicators that was selected at the start of the DHFA based on a systematic review of the available literature 16]. The QI-set is annually updated. Quality indicators can be divided in structural, process and outcome parameters. For this 5-year trend analysis four care process quality indicators and one outcome quality indicator were analysed.

Three of the four care process quality indicators were chosen because of international agreement on their positive effect on outcomes, according to several guidelines [17–19]. The quality indicator “Time to surgery” is met if the patient is operated within 48 h after arrival at the emergency department. The “Assessment for osteoporosis” indicator is met, if patients not already analysed for osteoporosis previous to the hip fracture, have a scheduled appointment for a bone mineral density measurement at moment of discharge. In the hip fracture population of 70 years and older, the “Orthogeriatric co-management” indicator is met if there is intensive co-treatment by the surgeons and geriatricians: the patient was either treated on a specialized geriatric-trauma ward or the geriatrician was consulted peri-operatively, not solely post-operatively. The fourth process indicator analyzed in this study is the “Registration of functional outcomes” indicator, which is used in the DHFA to gain insight into the level in which hospitals collect follow-up data on functional outcomes of their treated patients. This quality indicator is met if the level of mobility and activity in daily living is scored for the pre-fracture situation (baseline; scored at time of admittance) and after three months of follow-up in patients aged 70 and older who are alive after three months post-fracture. In the DHFA, the Fracture Mobility Score is used to measure the level of mobility and for activity in daily living the KATZ Index of Activities of Daily Living (KATZ-6 ADL) score is used [20, 21].

Case-mix corrected mortality within 30 days after admission was chosen as outcome quality indicator, since this is the most complete and objective outcome parameter in the DHFA at present.

### Other study parameters

Potential confounders/case-mix factors included in the analyses were age, gender, fracture side, fracture type, pre-fracture living situation, Fracture Mobility Score, KATZ-6 ADL score, American Society of Anesthesiologist physical status classification (ASA) score [22] and pre-fracture presence of dementia or osteoporosis. Risk of malnutrition was measured during hospital stay using the Short Nutritional Assessment Questionnaire (SNAQ) or the Malnutrition Universal Screening Tool (MUST) and categorized as low (SNAQ 0 or MUST 0), medium (SNAQ 1-2 or MUST 1) or high risk (SNAQ ≥ 3, MUST ≥ 2) [23, 24]. Baseline characteristics of included patients are reported using descriptive statistics.

### Statistical analysis

#### Trend over time for quality indicators

To determine if there was an independent association between quality indicator results and report year, mixed-effects logistic regression models were constructed with report year as fixed-effect factor and hospital as random-effect variable. The random effect per hospital was estimated separately for each year. For this trend analysis, report year 2016 was excluded due to the high possibility of selection bias as a consequence of the limited number of hospitals participating in this first year of registration. In the analysis of the outcome quality indicator “30-day mortality”, case-mix factors as noted above were added as fixed variables in the mixed-effect model.

Results are shown as probabilities (%) with 95% confidence intervals (CI). Differences between years were tested using Analysis of Variance (ANOVA). These mixed-effect models on trends over time per quality indicator were visually displayed in “spaghetti-plots”. In each plot, each thin line represents a hospital and shows the percentage in which the quality indicator is met (y-axis), per year (x-axis). The thick black line represents the overall probabilities with CI from the mixed-effect models.

Per quality indicator, cases with missing values needed for calculating the indicator score were excluded. Missing values for case-mix factors were imputed with the median value for continuous variables and with the mode for categorical variables. Due to the low case-numbers, having a bilateral fracture was not included as a case mix factor in the analysis of mortality. *P* values < 0.05 were regarded as statistically significant.

#### Between-hospital variation of quality indicators

The between-hospital variation of quality indicators is visually displayed with funnel plots per registration year. In the plots for the quality indicators “Time to surgery”, “Assessment for osteoporosis”, “Orthogeriatric co-management in patients ages 70 years and older” and “Registration of functional outcomes” the mean observed percentage is shown as the benchmark with the funnel representing the lower and upper bounds of its 95% confidence interval. For the quality indicator “30-day mortality” (Fig. 2e), we calculated the Observed/Expected ratio while taking a number of case mix variables into account when computing the expected frequency. This adjustment yields a standardized mortality ratio (SMR). Included case-mix factors included registration year in the DHFA, age, gender, fracture side, fracture type, pre-fracture mobility, pre-fracture independence (stratified as independent defined as KATZ6-ADL = 0 versus any form of dependency defined as KATZ6-ADL > 0), osteoporosis, ASA-score (stratified in 1–2 versus 3 or higher) and risk of malnutrition. In the funnel plot for “30-day mortality”, the benchmark equals SMR = 1.

Statistical analysis was performed using R Version 4.0.2 making use of the ‘lme4’ package for the linear mixed effects analysis and the ‘randomForest’ package for imputation of missing values [25–27].

## Results

### Data quality

**Fig. 1:**
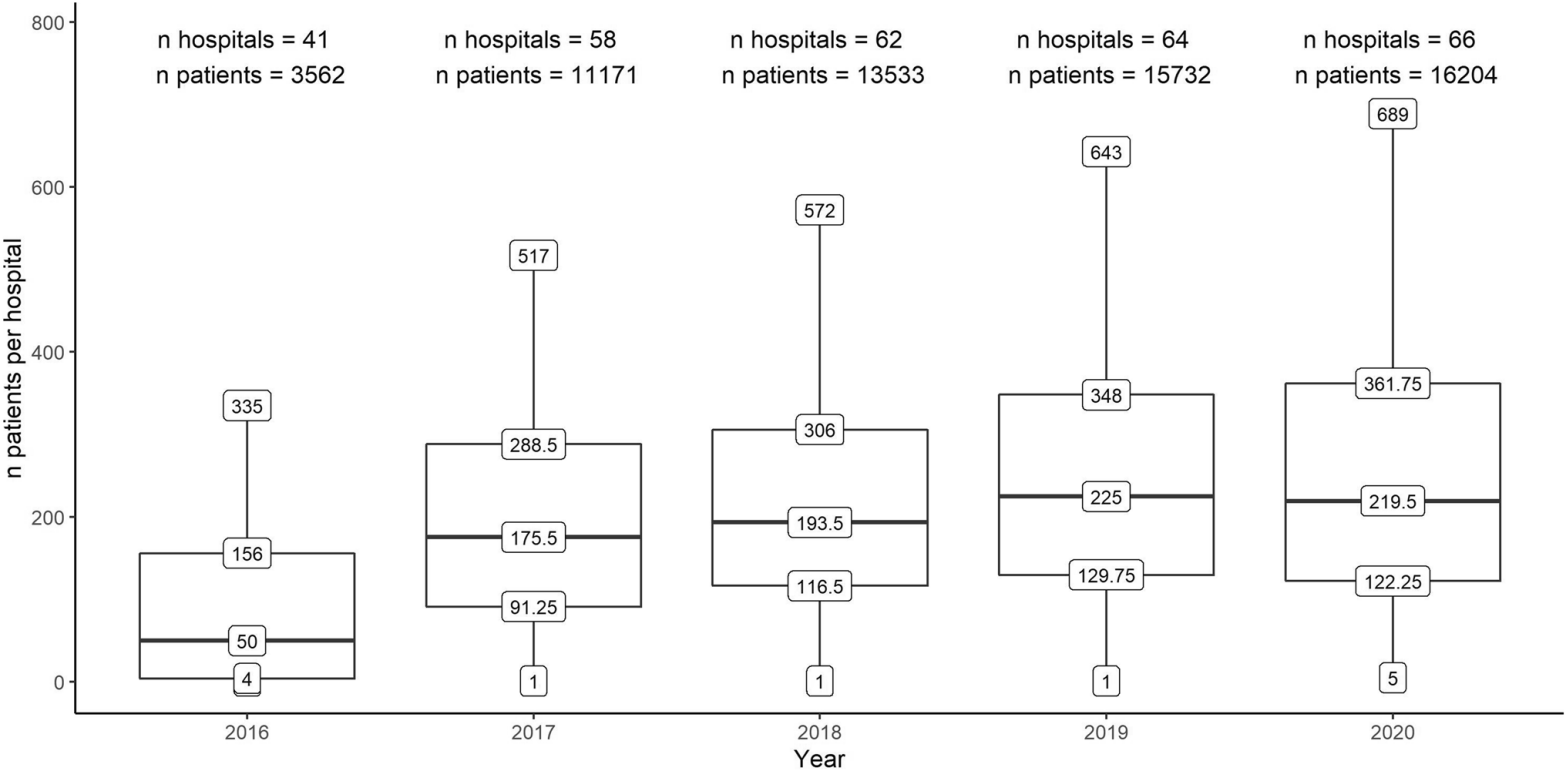
Annual number of participating hospitals and volume of patients in the Dutch Hip Fracture Audit from 2016 to 2020. The boxplots represent the median number of registered patients per hospital with the interquartile and total range

Between 01-01-2016 and 31-12-2020 a total of 60,202 patients were included in the DHFA. The number of participating hospitals per year and the numbers of patients recorded per hospital are shown in Fig. 1. The audit started in 2016 with 41 hospitals recording a median of 50 patients per hospital, and increased to 66 hospitals with a median of 220 patients registered per hospital in 2020. In 2020 the case capture was approximately 85% of all hip fracture patients in the Netherlands according to the Dutch Trauma Registry [5].

The percentage of missing data is shown in Table 1. The mean percentage of all missing values of variables gathered during the hospital admission decreased from 7.5% in 2016 to 3.2% in 2020. The data completeness of the 3-month follow-up data of the audit improved from 36.2 to 46.8%.

**Table 1:** Data completeness of the Dutch Hip Fracture Audit variables from 2016 to 2020

Year	2016	2017	2018	2019	2020
Total *n* patients	3562	11,171	13,533	15,732	16,204
	% missing	% missing	% missing	% missing	% missing
**Clinical section of survey**					
Date of birth	0.0	0.0	0.0	0.0	0.2
Gender	0.0	0.0	0.0	0.0	0.4
Fracture side	1.9	0.8	0.9	0.5	0.3
Pre-fracture living setting	8.4	19.2	10.4	11.4	11.4
Pre-fracture mobility score	5.8	14.9	10.7	2.1	0.9
Pre-fracture KATZ6-ADL	6.4	6.7	5.4	5.9	5.4
Dementia	10.7	16.4	10.4	12.5	7.9
Osteoporosis	12.6	20.8	14.6	17.7	13.1
Malnutrion scores in hospital	5.3	8.0	7.0	9.0	5.7
Specialty of physician in charge	4.1	13.4	3.4	0.8	1.8
Specialty of consulted physician	34.9	44.0	37.3	6.3	2.7
Time of stay on emergency Ward	12.8	16.8	10.8	9.6	5.8
Fracture type	12.2	18.2	9.8	1.3	1.1
Treatment type	2.2	2.4	2.0	1.3	2.3
Operation date and time^a^	0.5	0.2	1.0	0.2	0.0
ASA-score^a^	9.2	14.2	11.4	0.3	0.3
Type of anaesthesia^a^	6.3	14.0	11.1	11.7	3.8
Geriatric involvement^b^	7.6	12.1	4.7	1.5	0.6
Discharge date	7.2	13.4	7.1	5.9	2.5
Complications during admission	5.7	6.5	2.0	2.8	3.0
Deceased in hospital	9.7	2.2	0.6	0.2	0.6
**3-month follow-up section of survey**					
Deceased within 3 months	59.2	62.1	55.0	49.1	49.8
Date of follow-up consultation	58.7	57.4	50.9	46.7	48.0
Living setting at 3 months	60.9	63.5	56.3	51.9	51.9
Mobility score at 3 months	60.6	61.2	52.0	45.7	47.6
KATZ6-ADL at 3 months	64.8	65.2	57.6	51.2	51.2
Reoperation within 3 months^a^	55.7	59.0	50.1	43.3	43.4
Reason for reoperation^c^	1.6	1	0.3	1.6	0.0
Date of reoperation^c^	0.0	0.1	0.1	1.6	0.0
**1-year follow-up section of survey**					
Dead/alive status at 1 year^d^	93.3	88.7	87.2	89.8	98.4

*KATZ-6 ADL* KATZ index of activities of daily living [20], *ASA-score* American society of anesthesiologist physical status classification [22], *SNAQ* short nutritional assessment questionnaire [23], *MUST* malnutrition universal screening tool [24]

^a ^% of operatively treated patients

^b ^% of patients ≥ 70 years old

^c ^% of patients registered as reoperated on within three months

^d ^% of patients alive after three months, based on data of the Vektis institute

Regarding data accuracy, comparison of the dates of death entered in the DHFA with the dates of death from Vektis was possible in 876 cases. In 843 (96.0% of 876) patients, the reported date in the DHFA corresponded with the date of death reported by Vektis (with a ± 1 day tolerance).

### Baseline characteristics

Baseline characteristics of patients recorded in the DHFA per year are shown in Table 2. The median age of registered hip fracture patients was 82 years (IQR 73–88), and the majority were female (66.6%). Most patients were treated operatively under spinal anesthesia; 2.8% of patients were treated conservatively. 46.3% of patients were mobile without needing mobility aids prior to admission, 44.9% were dependent in activities of daily living and the majority was living at home before the fracture (77.0%). Almost 1 out of 5 patients was reported to have dementia (19.2%), and 1 out of 8 was already diagnosed with osteoporosis (12.1%). The majority of patients had an ASA-score of III or higher (58.0%).

**Table 2:** Baseline characteristics of patients included in the Dutch Hip Fracture Audit from 2016 to 2020

Year	2016	2017	2018	2019	2020	Total
Total number of patients per year	3562	11,171	13,533	15,732	16,204	60,202
Age (median)	82	82	82	81	81	82
Female gender (%)	68.9	67.0	66.2	67.2	65.7	66.6
Fracture side (%)						
Right	47.9	48.1	48.2	47.9	47.9	48.0
Left	52.1	51.8	51.7	52.1	52.1	51.9
Bilateral	0.1	0.1	0.1	0.0	0.0	0.1
Fracture type (%)						
Femoral neck, undislocated	16.3	17.6	18.2	17.7	16.8	17.5
Femoral neck, dislocated	39.0	38.4	37.4	36.9	38.6	37.8
Trochanteric type AO-A1	12.9	13.8	13.9	13.8	13.3	13.6
Trochanteric type AO-A2	24.7	20.4	19.0	19.0	18.5	19.4
Trochanteric type AO-A3	6.3	5.7	6.0	5.8	5.7	5.8
Subtrochanteric	0.4	2.6	3.3	4.1	4.4	3.6
Unspecified	0.4	1.6	2.2	2.7	2.7	2.3
Type of anaesthesia (%)						
General	38.9	35.6	36.2	38.1	39.5	37.7
Regional	0.9	0.5	0.5	0.7	2.5	1.1
Spinal	55.7	53.4	54.2	50.4	47.4	51.3
General + regional	2.5	2.8	2.4	3.6	3.3	3.0
General + spinal	0.5	2.0	0.8	0.8	0.9	1.0
Spinal + regional	1.5	5.7	5.9	6.3	6.2	5.8
Spinal + regional + general	0.0	0.0	0.0	0.1	0.1	0.1
Treatment type (%)						
Conservative	2.0	2.0	2.7	2.8	3.7	2.8
Hemiarthroplasty	35.0	35.3	34.5	33.3	34.5	34.4
Cannulated screws	6.4	5.9	6.0	5.2	4.3	5.3
Total hip arthroplasty	4.0	4.3	5.6	6.9	6.9	6.0
Sliding hip screw	11.3	13.2	12.7	13.2	13.5	13.1
Intramedullary nailing	41.2	39.2	38.5	38.6	37.0	38.4
Girdle stone	0.0	0.0	0.0	0.0	0.1	0.0
Pre-fracture mobility (%)						
Unknown	5.3	8.6	6.6	6.6	5.3	6.5
Not using any mobility aid	47.9	44.4	45.7	46.6	47.3	46.3
Mobile outdoors using 1 mobility aid	6.5	5.7	5.6	6.1	7.3	6.3
Mobile outdoors with 2 aids or frame	30.7	31.3	30.3	30.8	30.8	30.8
Mobile indoors but never outside without help of others	7.3	7.7	8.5	7.2	7.8	7.7
No functional mobility (no use of lower extremities)	2.3	2.2	3.4	2.6	1.5	2.4
Dependent in activities of daily living (KATZ6-ADL > 0) (%)	45.1	46.2	45	45.2	43.5	44.9
Pre-fracture living situation (%)						
At home	73.6	75.3	76.5	77.6	78.9	77.0
Institutionalized	24.4	22.3	20.8	19.7	18.8	20.4
Unknown	2.0	2.4	2.7	2.8	2.3	2.5
Known with dementia (%)	21.6	20.9	19.4	18.4	18.1	19.2
Known with osteoporosis (%)	13.5	12.9	11.8	11.9	11.8	12.1
ASA-score III, IV or IV (%)	55.1	57.8	57.1	58.2	59.3	58.0
Risk of malnutrition (%)						
No risk (SNAQ 0 or MUST 0)	85.6	86.3	84.7	86.0	86.1	85.8
Medium risk (SNAQ 1–2 or MUST 1)	3.1	3.4	4.1	4.1	3.9	3.9
High risk (SNAQ ≥ 3, MUST ≥ 2)	11.3	10.4	11.2	9.9	10.0	10.4

*KATZ-6 ADL* KATZ index of activities of daily living [20], *ASA-score* American society of anesthesiologist physical status classification [22], *SNAQ* short nutritional assessment questionnaire [23], MUST malnutrition universal screening tool [24]

### Time to surgery

**Fig. 2:**
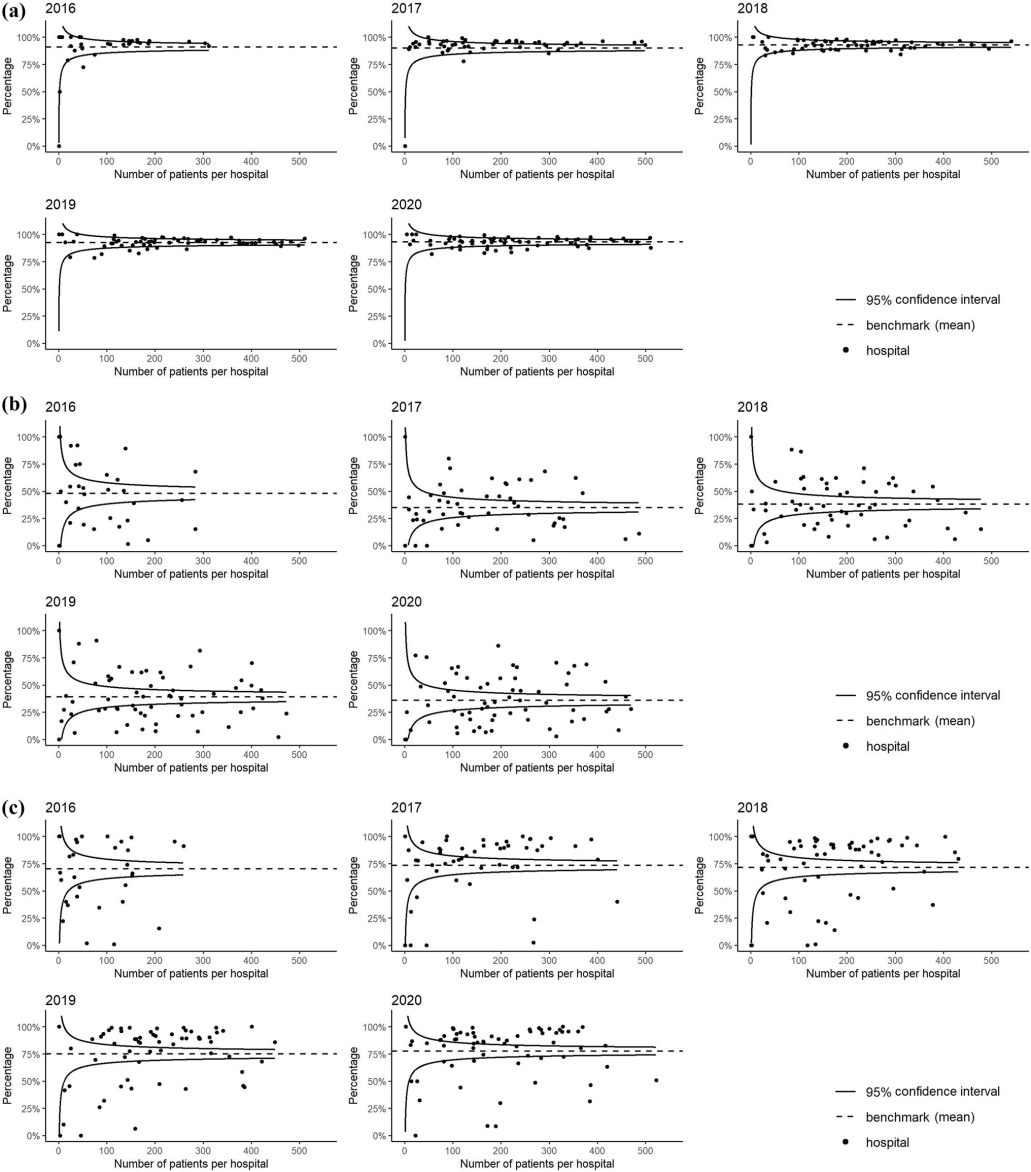

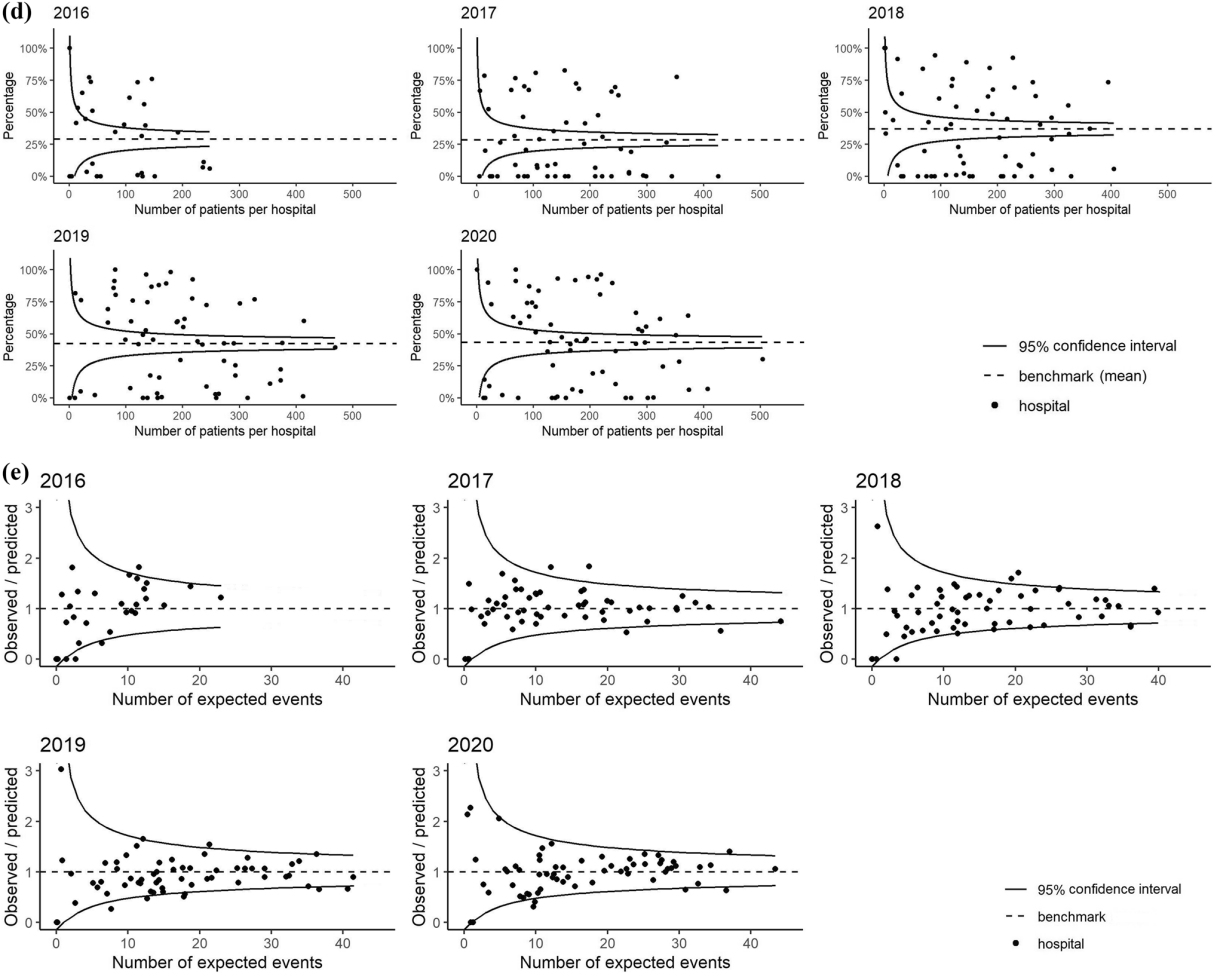
Funnelplots of between-hospital variation in the percentage of meeting quality indicators (QI) for hip fracture care from 2016 to 2020. **a** QI: Operation within 48 h, **b** QI: Assessment of osteoporosis, **c** QI: Orthogeriatric co-management in patients aged 70 years and older, **d** QI: Registration of functional outcomes at three months in patients aged 70 years and older, **e** QI: Case-mix adjusted 30-day mortality (expressed as standardized mortality ratio); adjusted for: age, gender, fracture side, fracture type, pre-fracture mobility, pre-fracture independence, osteoporosis, ASA-score and risk of malnutrition

**Fig. 3:**
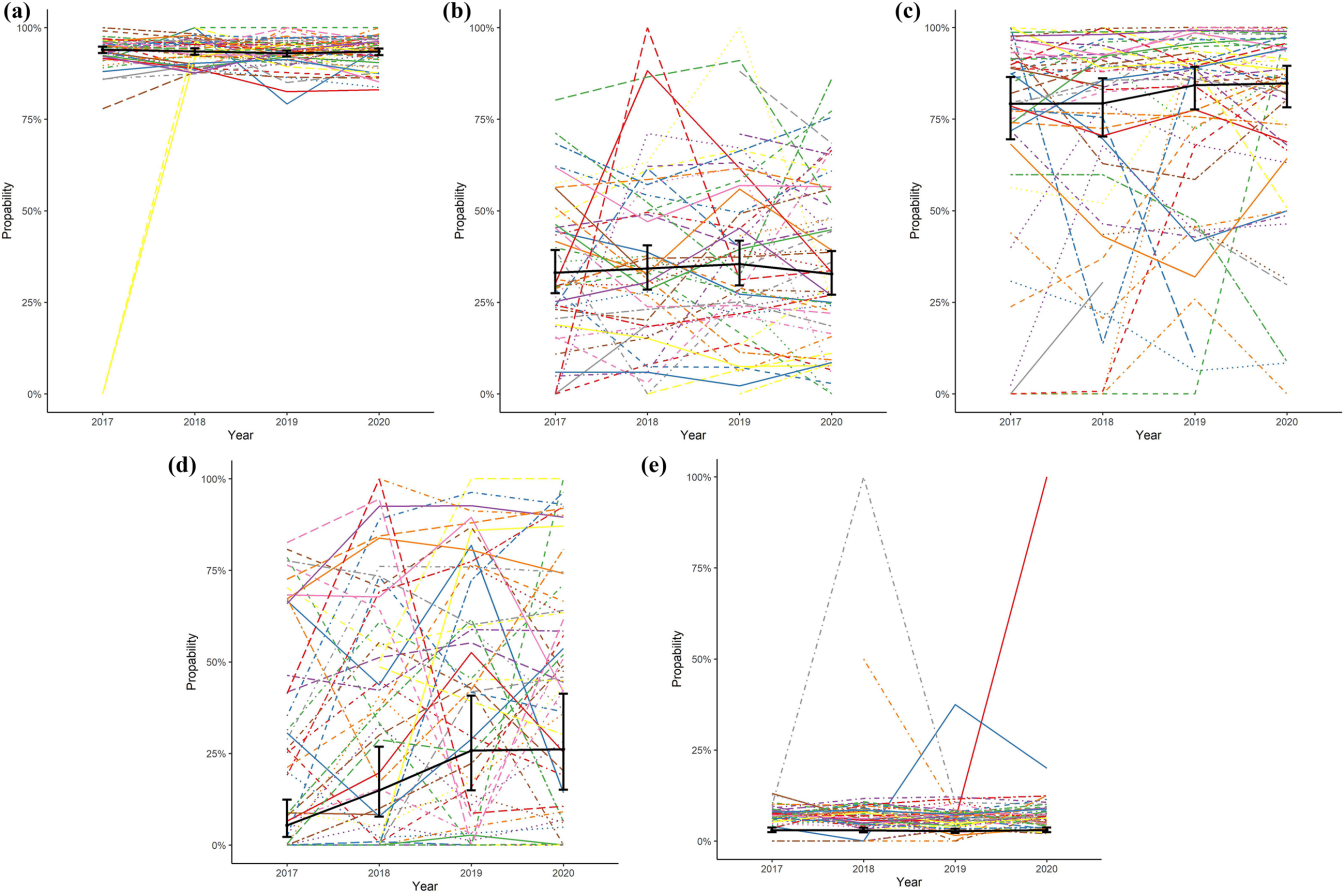
Trends in time for the probability of meeting quality indicators (QI) for hip fracture care. Coloured lines represent the percentages of patients meeting the QI per hospital per year. The solid black line represents the mean probabilities of meeting the QI per year with 95% confidence interval. **a** QI: Operation within 48 h, **b** QI: Assessment for osteoporosis, **c** QI: Orthogeriatric co-management in patients 70 years and older, **d** QI: Registration of functional outcomes at three months in patients 70 years and older, **e** QI: Case-mix adjusted 30-day mortality

Out of the 57,429 operatively treated patients, 816 were excluded from analysis of this QI due to missing data. The mean observed hospital percentage meeting this quality indicator was 91.1% in 2016 and 93.0% in 2020 (Fig. 2a). There was limited between-hospital variation in the percentage of patients operated within 48 h for each registration year (Fig. 2a). The mixed-model (Table 3 and Fig. 3a) showed no statistically significant change in the patients’ probability of being operated within 48 h between registration years (*p* = 0.16).

**Table 3:** Results of mixed-model analysis for trends in hip fracture quality indicators in period 2017–2020

	Probability (%)	95%-CI	ANOVA *p* value of year effect
Time to surgery (< 48 h)			0.16
2017	94.0	93.1–94.8%	
2018	93.5	92.6–94.3%	
2019	93.0	92.2–93.7%	
2020	93.4	92.5–94.3%	
Assessment for osteoporosis			0.68
2017	33.1	27.5–39.3%	
2018	34.3	28.5–40.5%	
2019	35.5	29.6–41.8%	
2020	32.8	27.1–39.0%	
Orthogeriatric co-management in patients aged 70 and older			0.51
2017	79.2	69.5–86.4%	
2018	79.3	70.2–86.1%	
2019	84.3	77.6–89.2%	
2020	84.7	78.2–89.6%	
Registration of functional outcomes in patients aged 70 and older			** < 0.01**
2017	5.4	2.2–12.4%	
2018	15.0	7.8–26.8%	
2019	25.8	15.0–40.8%	
2020	26.1	15.1–41.3%	
Case-mix adjusted* 30-day mortality			
2017	3.0	2.4–3.7%	0.21
2018	3.0	2.4–3.7%	
2019	2.7	2.2–3.4%	
2020	3.0	2.4–3.7%	

*ASA-score* American society of anesthesiologist physical status classification [22]

*Adjusted for: Age, gender, fracture side, fracture type, pre-fracture mobility, pre-fracture independence, osteoporosis, ASA-score and risk of malnutrition. Reference categories used are: Female gender, left-sided fracture, trochanteric AO-A2 fracture type, mobile outdoors with 2 aids or frame, independent in daily living activities, ASA-score 3,4 or 5, and no risk of malnutrition

### Assessment for osteoporosis

In total, 46,666 patients were not analysed for osteoporosis previous to the hip fracture and therefore eligible for analysis of assessment for osteoporosis. The mean observed percentage of these patients meeting this quality indicator in 2016 was 48.0%, and in 2020 this percentage was 35.8% (Fig. 2b). The funnel plots in Fig. 2b show that there was wide between-hospital variation in the percentage of patients with assessment for osteoporosis for each registration year, with a large number of hospitals scoring higher or lower than the benchmark (i.e., outside the funnel). In 2020 the best performing hospital scored 86.1%. The mixed model (Table 3 and Fig. 3b) showed no change in the patients’ probability of having assessment for osteoporosis (*p* = 0.68) over the years.

### Orthogeriatric co-management in patients aged 70 and older

Of 49,114 patients aged 70 years and older, 46,529 were eligble for analysis of orthogeriatric co-management. The mean observed percentage of patients in which this quality indicator was met was 70.1% in 2016 and 77.6% in 2020 (Fig. 2c). Figure 2c shows that the percentage of patients 70 years and older with orthogeriatric co-management varied from 0 to 100% between hospitals for each registration year. Although the patients’ probability of orthogeriatric co-management increased slightly over the years (Table 3 and Fig. 3c), this trend was not statistically significant in the mixed model (*p* = 0.51).

### Registration of functional outcomes in patients aged 70 and older

43,855 patients were aged ≥ 70 years and thus eligible for analysis of registration of functional outcomes. In 2016 the mean observed percentage of these patients with registered functional outcomes was 28.8%, while this was 43.2% in 2020 (Fig. 2d). For each registration year, the percentage of patients meeting this QI varied from 0 to 100% between hospitals (Fig. 2d). Despite the ongoing wide between-hospital variation for this QI, the mixed-model (Table 3 and Fig. 3d) showed a statistically significant increase in the patients’ probability of registration of functional outcomes (5.4% in 2016 versus 26.1% in 2020, *p* < 0.01).

### Case-mix adjusted 30-day mortality

Survival status was available for 57,571 patients. The between-hospital variation in casemix-adjusted 30-day mortality from 2016 until 2020, as shown in Fig. 2e, was limited. Each year, the 30-day mortality in only one or two hospitals was statistically higher or lower than expected based on their casemix (SMR exceeding the upper or lower boundary in the funnel plots in Fig. 2e). The mixed-effect model (Table 3 and Fig. 3e) with case-mix adjustment showed no significant changes in the 30-day mortality over the years (*p* = 0.21).

## Discussion

After implementation of the Dutch Hip Fracture Audit in 2016, the DHFA has had an increased capture rate in hospitals and in the number of patients. Currently, 66 out of 68 hospitals in The Netherlands treating hip fracture patients participate, and approximately 85% of all hip fracture patients are registered within the DHFA [5]. Over the last 5 years the data quality has improved. Scores on the QI time to surgery remained unchangingly high, assessment for osteoporosis was low and did not significantly improve, orthogeriatric co-management increased somewhat but not with statistical significance, registration of functional outcomes improved significantly and case-mix adjusted 30-day mortality rates remained unchanged.

The data completeness improved over the last five years: the average percentage of missing values in the registries clinical section dropped from 7.5% in 2016 to 3.2% in 2020, and the 3 months follow-up completeness increased from 36.2% in 2016 to 46.8% in 2020. The rise in completeness of follow-up data is likely a result of the Dutch Health Inspectorate mandating registration of functional outcomes since 2020. Still, we believe the remainder of missing values, especially in the outcome variables, to be selective, as the reported in-hospital complication and reoperation rates (not presented in this study) appear lower when compared to other registries or studies. Dutch data protection legislation hinders direct extraction of outcome information from valuable sources such as social care institutes after the patient is discharged.

The linkage of the DHFA with a database from the Vektis institute however, has solved a significant reporting bias for mortality, resulting in mortality rates in expected ranges. After linking the Vektis-data with the DHFA accurate survival status of 95.6% of recorded patients was achieved. The unadjusted 90-day mortality rate based on merely DHFA data was 4.2% and after linking DHFA data with Vektis data this rate significantly increased to 12.5%. Although the number of missing dates of death in the DHFA is considerable, data accuracy of entered dates of death was high (96.0%). Given the variation in data completeness per hospital, per patient and per variable, data quality continues to be a priority for the DHFA. Data verification or audit filters described by other registries could be used as examples for further improvement [7, 28–30].

The median age of the registered hip fracture patients was 82 years, and 66.6% of the patients were female. These characteristics as well as fracture types and pre-fracture living settings are comparable with the baseline characteristics of hip fracture populations in other European registries presented in a recent systematic review [31]. Notably, the proportion of patients with ASA-scores ≥ 3 is low in the Netherlands (58.0% versus range 61–74.5%). Also, the proportion of patients that is only mobile inside their house, is low (7.7%, versus range 10–28.3%) [31]. The only patient characteristic in the DHFA that changed over time is the pre-fracture living situation: 73.6% lived independently at home prior to the fracture in 2016 compared to 78.9% in 2020. The Dutch Trauma Registry also showed this trend [5].

Over the last 5 years 2.8% of the hip fracture patients was treated conservatively, which is comparable with other countries. However, our data show a slight increase in percentage of patients treated conservatively when comparing 2016–2020 (2.0% versus 3.7%). This may be due to a selection bias in early registration years. Or it may reflect a true numerical effect, as attention for the natural course due to other co morbidities in terminal patients with a hip fracture, and the so-called end of life planning, seems to be growing. Studies on non-operative treatment in frail institutionalized patients underline this interest [31, 32].

The results for the quality indicator time to surgery within 48 h after presentation at the emergency department did not change over time. This is likely due to the fact that this quality indicator was already met in more than 90% of cases at the start of the DHFA in 2016, leaving little room for improvement thereafter. Our percentages are comparable with percentages provided by registries of USA, Sweden, Denmark and Germany [6], which could indicate that the highest level of performance in this quality indicator is already met. However, in 2020, eleven hospitals were still performing significantly less well than the overall hospital mean on this quality indicator (hospitals below the 95%-CI line in the funnel plot in Fig. 2a). This may result from these hospitals’ case-mix, which we were unable to adjust for, due to the lack of information on relevant parameters (especially use of direct oral anticoagulants). Alternatively, it may reflect the hospitals’ actual processes. This should therefore be further analyzed.

The scores on the indicator assessment for osteoporosis did not significantly increase over time. The mean percentage of patients per hospital in which this quality indicator is met, was only 35.8%. This is a worrisome finding as from a series of twelve hip fracture registries, only two other countries show such low percentages [6]. The between-hospital variation is high; the highest performing hospital scored 86.1%, showing high percentages are achievable. However, the current definition of this DHFA quality indicator leaves room for interpretation. This quality indicator registers if a DXA-scan is scheduled when the patient is discharged from the hospital; it is unclear whether this scan is actually performed and if the patient had follow-up treatment thereafter. Therefore, these percentages may even be an overestimation of the diagnostic process for osteoporosis. Also, the high number of missing values on osteoporosis screening asks for interpreting these results with caution. Since a revision of the Dutch guideline on osteoporosis care is currently ongoing, this quality indicator will need to be reviewed and probably revised to better mirror the osteoporosis treatment and fracture prevention given to hip fracture patients in the Netherlands.

The performance on the quality indicator orthogeriatric co-management has slightly improved over the last five years, but not enough to show a statistically significant trend in our analysis. Most hip fracture registries report percentages of patients reviewed by a geriatrician between 69 and 91%, of which six out of eight reported percentages higher than our result of 77.6% in 2020 [6]. However, it is unclear at which point in the care pathway these reported geriatric reviews took place or to what degree the geriatrician was involved. Thus, the orthogeriatric co-management percentages of other registries can also represent a short post-operative consultation, which is not recommended in the Dutch guidelines. The Dutch guidelines recommend having a geriatrician at least involved pre-operatively 19, 33]. It is not sure if the Dutch mean score on this quality indicator is comparable to the international percentages. A recent meta-analysis showed significant positive effects of any model for orthogeriatric care (in which surgeon and geriatrician collaborated): a decrease in hospital length of stay of 1.55 days, a 28 and 19% lower risk of in-hospital mortality and 1-year mortality and a 19% lower risk of delirium [34]. Still, in 2020 as many as 19 (31.1%) hospitals performed significantly less well than the mean performance of all Dutch hospitals (hospitals scoring below the 95%-CI line in the funnel plot in Fig. 2c), indicating very wide between-hospital variation. In contrast to the time to surgery and osteoporosis quality indicators, the results for this quality indicator cannot be biased by the hospitals’ patient case-mix: outlier hospitals are genuine outliers. This obviously indicates that there is room for improved orthogeriatric co-involvement in one third of the hospitals in the Netherlands.

The authors believe that the value of a registry is the highest when there is sufficient outcome data available: only in this way the effect of processes can be evaluated. Remarkably, international comparative studies on hip fracture registries do not mention functional outcomes [6, 13, 31, 35]. Even more surprising: a recent review on hip fracture quality indicators did not include functional outcomes in their recommendations [36]. The probable reason is the difficulty in collecting this information, which appears to be a struggle for most hip fracture registries [7]. A trends analysis from the Swedish registry reported unchanged functional outcomes in hip fracture patients over the past 25 years, likely due to the worsened medical condition and increasing age in registered patients, which may counteract the improvements in the care processes [37]. Besides this study, large cohorts reporting functional outcomes are lacking. Fortunately, a significant increase is seen in the availability of functional outcomes within the DHFA registry. The mean percentage increased from 28.8% in 2016 to 43.2% in 2020. In 2020 the Dutch Health Care Inspectorate recognized the importance of functional outcome and made the registration of functional outcomes a mandatory quality indicator, which is likely to have contributed to the improvement of registration of the 3 months follow-up data on functional outcomes and probably will to lead to ongoing completeness of functional outcome data in the future.

The between-hospital funnel plots in Fig. 2d show that hospitals are capable of adequately collecting functional outcomes, despite having high numbers of patients per year. Sharing best practices in collecting functional outcomes is also a promising tool to further increase the data quality of these parameters, so these can be used in future benchmarking of quality of care, both in the Netherlands and internationally. Besides, insight in functional outcomes may help in shared decision making in the daily clinical practice in hip fracture care.

According to this DHFA-data analysis, the 30-day mortality rates have not significantly changed over the last five years. Previous studies on this topic seem to come to varying conclusions. A meta-analysis published in 2019 reports a decrease in 1-year mortality and ascribes this result to hip fracture registries [38]. Other studies show the mortality rates for hip fracture patients have not improved, despite the increased number of hip fracture registrations and evaluation of care [36, 39]. All registry studies report unadjusted mortality rates or do not show trends in 30-day mortality over the years, which hinders international comparisons of the trends in mortality. Mortality was chosen as a quality indicator as it is an objective and accurate parameter, especially since the link of the DHFA with Vektis data. Nonetheless, we should question whether this is the best outcome measure for hip fracture care; it could be better to focus on the patient’s functional outcomes as one might assume that for many elderly patients self-sustainability is far more important than life-duration.

### Strengths and limitations

As in many studies that use data of registries, the main limitations concern the fact that the data could not be validated and that the number of missing values is considerable.

The trends over the years may have been influenced by differences in numbers of included hospital and their data quality. This leads to a risk of selection bias, especially in the first registration years. In 2016, only 41 hospitals participated and 3562 patients were included. We therefore advocate that this analysis is repeated when the inclusion of patients and hospitals has been on a standard level for several years. To minimize the effects of selection bias, data of the year 2016 was excluded from the mixed-model trend analyses. As seen in the trend Fig. 3a–d; some hospitals’ performances vary over the years and it is unsure whether this is due to registration errors or realistic hospital performance.

Another limitation is the limited amount of clinical outcome data; due to the use of Vektis-data the most trustworthy clinical outcome parameter is mortality, which is not always the most interesting outcome when hip fracture patients are concerned. The results on the quality indicator of registration of functional outcomes reflect the difficulty of collecting outcome data of hip fracture patients, although we have shown the promisingly increasing trend in registration as well as hospitals with high scores on this indicator.

Lastly, trends in data cannot be directly ascribed to the Dutch Hip Fracture Audit, however trends may be influenced by the registry as it provides mirror information for the participating hospitals.

### Future perspectives

The results of this study point out where there is room for improvement on specific quality indicators. As said the main limitation of quality of care registries as the DHFA, is that their value depends on the data quality. The data on care processes in the DHFA are considered sufficiently complete, but data completeness of outcomes needs to be improved. Linkage with another database supplemented the DHFA with accurate mortality data. Besides complementing the data from an external source, another benefit of linking a registry with a trustworthy external data source is that it enables validation of parameters that are present in both datasets. The authors believe linkage between datasets would be the way to increase the value of clinical registries for quality of care registries in general. The possibilities for linkage of datasets under the Dutch privacy laws are currently very limited, therefore updated privacy legislation regarding linking datasets for quality of care purposes is required.

To improve data quality and to optimize the use of the registry in the future, several projects have been started within the DHFA. Data verification of selected DHFA parameters using data of reimbursed medical costs as a reference is being carried out which will give insight into the quality of specific data in the DHFA-, such as in-hospital complications and reoperations. Also, a common care pathway for hip fracture patients in all electronical patient file software systems is due to be implemented. This eventually will result in automated registration of all hip fracture patients within the DHFA lowering the burden of registration and improving data quality. In order to get better information on functional recovery, a pilot-project on patient reported outcome measures (PROMs) has been carried out. This project hopefully will answer the question if the use of PROMs is feasible for gaining information on recovery in this elderly population. Another pilot-project tested new variables on their potential for case-mix correction, resulting in data driven improvement of the DHFA. Lastly, the improvement of the interactive ‘Codman Dashboard’ which provides hospitals with their data and national benchmarks, will hopefully lead to increased use of the already available mirror information for hospitals to improve their hip fracture care. Eventually, when the registry has matured, data is validated, and scores on designated quality indicators are persistently high, one could work towards Value-Based Healthcare: striving to maximize health outcomes for the patient whilst lowering health care costs.

## Conclusion

In the past 5 years, the Dutch Hip Fracture Audit has been successfully implemented, with 66 out of 68 Dutch hospitals participating, and covering 85% of the hip fracture patients in the Netherlands. The data completeness has improved over the years, especially in the clinical section of the registry, as well as concerning information on mortality rates. However, data verification still has to become standard procedure and the data quality of functional outcomes needs to be improved. The quality indicators show persistent high performances over the years concerning time to surgery within 48 h. The average performance on orthogeriatric co-management has improved, but hospitals show an unchanged poor performance on the assessment for osteoporosis, with wide between-hospital variation. The 30-day mortality of Dutch hip fracture patients has remained unchanged and the DHFA presents a growing insight in functional outcomes at three months after the fracture.

Improvements such as automated registration, pilot projects on PROMS, datavalidation and new quality indicators together with an interactive Codman Dashboard are initiated to optimize the potential of the DHFA, and hopefully as a result, to optimize hip fracture care.
